# Linear and Curvilinear Relationship between Knee Range of Motion and Physical Functioning in People with Knee Osteoarthritis: A Cross-Sectional Study

**DOI:** 10.1371/journal.pone.0076173

**Published:** 2013-09-26

**Authors:** Thomas J. Hoogeboom, Nico L. U. van Meeteren, Raymond H. Kim, Jennifer E. Stevens-Lapsley

**Affiliations:** 1 Physical Therapy Program, Department of Physical Medicine and Rehabilitation, University of Colorado, Denver, Colorado, United States of America; 2 Caphri Research School, Maastricht University Medical Centre, Maastricht, The Netherlands; 3 Center for Care Technology Research (CCTR), Maastricht, The Netherlands; 4 TNO Healthy Living, Leiden, The Netherlands; 5 Department of Orthopedics, Porter Adventist Hospital, Denver, Colorado, United States of America; University of Texas Health Science Center at Houston, United States of America

## Abstract

**Background:**

Knee range of motion (KROM) is associated with the ability to perform daily activities in people with knee OA. However, this association is weak, possibly through the use of linear analyses. Curvilinear associations appear much more relevant, as these allow the determination of relevant clinical thresholds in KROM. The goal of this study is to assess the curvilinear association between KROM and daily activities (self-reported and observed) in people with knee osteoarthritis (OA).

**Methods:**

Demographic, functional and KROM (flexion and extension) data were collected from a convenience sample of people with knee OA awaiting total knee arthroplasty. Self-reported functioning was measured by use of the Knee Osteoarthritis Outcomes Scale and observed functioning with the timed up and go and six-minute walk test. The presence of curvilinear relationships between KROM and measures of functioning were tested by generalized additive modeling, piecewise regression modeling and receiver operated curves.

**Results:**

Data from 110 participants (mean age ± standard deviation: 65 ± 9 and female: 54%) with knee OA were evaluated. Statistical modeling did not reveal linear nor curvilinear associations between KROM and self-reported or observed measures of functioning; except for statistical significant associations between reduced knee flexion and major difficulties standing (*p*<=0.01). However, further modeling did not provide convincing evidence for relevant clinical associations and thresholds.

**Conclusions:**

No clinically relevant relationship between KROM and self-reported or observed measures of physical functioning could be established, indicating that the limitations in range of motion in the affected knee OA alone do not contribute to poorer functional performance.

## Introduction

Functional disability is a typical characteristic in people with knee osteoarthritis (OA) [1]. Over the years, numerous factors have been proposed as possible explanations for the level of functional disability in people with knee OA [2,3]. Among these factors is reduced knee joint range of motion (KROM) [4,5]. However, the association between KROM and functional disability is only weak to moderate [4–6]. We hypothesize that this weak association is a result of using traditional correlational analyses, which test the strength of linear associations between KROM and functional disability [7]. Such a linear relationship would suggest that any improvement in KROM will lead to some improvement in function [8], this seems unlikely. Perhaps a model studying KROM and functional disability could be improved by introducing curvilinear-terms [8]. This information could assist clinicians by providing evidence on the minimum level of KROM above which an increase in KROM does not translate into clinically important improvement in function (i.e. the identification of clinically relevant thresholds).

To our knowledge, no prior studies have investigated the additive value of adding curvilinear-terms to a statistical model describing the relationship between KROM and functional disability in people with knee OA. This is remarkable because the identification of clinical thresholds through the association between KROM and functional disability in people with knee OA could provide invaluable information to guide physical therapy treatment priorities. Therefore, the purpose of this exploratory study was to assess curvilinear association between KROM and functional disability (self-reported and observed) and to evaluate the potential for clinically relevant thresholds in KROM in people with knee OA.

## Methods

In this cross-sectional study, we used a convenience sample of preoperative baseline data that were collected for two randomized clinical trials from June 2006 to June 2010 at the University of Colorado Hospital [9,10]. Patients were scheduled for primary, unilateral total knee arthroplasty (TKA) for OA and had (1) to be between 50 and 85 years old, (2) no significant neurologic impairments (2), no uncontrolled hypertension, (3) minimal contralateral knee OA (as defined by pain during activity <5 out of 10), (4) no other unstable lower-extremity orthopedic conditions, (5) a body mass index (BMI) of 40 kg/m^2^ or less. Note: for one of the clinical trials (n=44) an additional inclusion criterion was a minimum of 80° of active knee flexion, however, nobody was actually excluded on the basis of this criterion [9]. Both studies were approved by the Colorado Multiple Institutional Review Board and informed consent was obtained from all participants.

### Outcome measures

Demographic characteristics were assessed by standardized, straight-forward questions to the patients. Pain, stiffness, and health-related quality of life were assessed with the validated and reliable Knee Osteoarthritis Outcome Score (KOOS) [11]. Functional disability was assessed by self-report and performance-based outcome measures. Self-reported functional disability was determined with the KOOS on both a composite score (i.e., KOOS subscales daily activities and sports and recreational activities) and activity score (individual activities scored as None / Mild / Moderate / Severe / Extreme difficulty). Patients’ opinion concerning 19 activities were studied, comprising 15 daily activities (descending stairs, ascending stairs, rising from sitting, standing, bending to the floor and picking up an object, walking on a flat surface, getting in/out of a car, putting on socks/stockings, rising from bed, taking off socks/stockings, lying in bed (turning over, maintaining knee position), getting in/out of bath, sitting, getting on/off toilet) and five sports/recreational activities (i.e., squatting, running, jumping, turning/twisting on injured knee, and kneeling). All KOOS subscale scores were transformed to a 0–100 scale, i.e., 100 – [actual raw score x 100 / possible raw score range], where lower scores represent unfavorable outcomes. Functional performance-based measures included the Timed Up & Go Test (TUG) and the six-minute walk test (6MWT). The TUG measures the time to rise from an armchair, walk 3 meters, turn around, and return to sitting in the same chair without physical assistance [12,13]. The 6MWT measures the total distance walked (in meters) over 6 minutes. This test has been used extensively to measure endurance and has been validated as a measure of functional mobility following TKA [13,14].

Finally, active KROM was measured in the supine position using a long-arm goniometer as previously described [15]. For active knee extension, the heel was placed on a 10 centimeter block, and the participant was instructed to actively extend the knee. For active knee flexion, the participant was instructed to actively flex the knee as far as possible while keeping the heel on the supporting surface. Throughout this manuscript, negative values of extension represent hyperextension [10].

### Statistical analyses

Descriptive statistics were used to describe the number of missing data and the study population. To determine the relationship between KROM and performance-based and self-reported composite measures of functioning, we used the statistical methods described by Ferrucci et al [16]. We first examined the independent effects of age, sex and BMI on walking distance (6MWT), functional mobility (TUG) and KOOS subscale Daily Activities and Sport and Recreational Activities in linear regression models, as the literature suggests these factors are associated [2,3]. Formal tests for differences between the linear fit and the local regression smoother fit were performed using nonparametric generalized additive models (GAM) in which the linear function for KROM was replaced by a locally weighted regression smoother (cubic splines with df=4) [16,17]. If GAM yielded p<0.10, the values of KROM-defining intersection points between segments specified in these models were further investigated by use of piecewise regression modeling.

To predict what percent of people report major difficulties for each of the 19 individual activities on the basis of KROM, we used logistic regression modeling. The response for each activity was dichotomized by grouping people as having *little difficulty* or *major difficulty*, by collapsing ‘none, mild or moderate difficulty’ and ‘severe or extreme difficulty’ for the daily activities and ‘none, mild, moderate or severe difficulty’ and ‘extreme difficulty’ of the sports/recreational activities. The difference in dichotomization between daily activities and sports/recreational activities was deemed necessary because daily activity scores are reported as substantially less difficult. Receiver operator curves (ROC) were plotted for those activities that the logistic model yielded p<0.10. ROCs that yielded an Area Under the Curve (AUC) of 0.70 or higher were studied in detail by describing sensitivity and specificity for the ideal KROM cut-off value. Further, all analyses were performed based on complete case data and all statistical analyses were carried out using statistical package Stata/IC 12.

## Results

We used data of 110 people (mean age ± SD: 65 ± 9; 54% female) with knee OA. KROM flexion and extension ranged from 80° to 143° and -8° to 25°, respectively. See Table 1 for detail on the characteristics of the study population. The majority of cases had no missing data (92%). Variables that had the most missing data were the KOOS item-activities: Getting in and out of bath (n = 6; 6%) and Getting in and out of a car (n = 5; 5%).

**Table 1:** Characteristics of study participants.

			N	Mean (SD)*
Age, in years			110	65 (9)
Sex, n (%♀)			110	59 (54)
BMI, in kg/m^2^			110	30 (5)
Timed up and go test, in sec, median (IQR)			109	8.3 (6.7-10.6)
Six-minute walk test, in meters			108	432 (124)
KOOS^†^				
		Pain (0-100)	106	50 (14)
		Symptoms (0-100)	106	51 (17)
		Daily activities (0-100)	106	59 (16)
		Sport and recreational activities (0-100), median (IQR)	106	15 (5-30)
		Quality of life (0-100), median (IQR)	106	25 (13-38)
Knee range of motion				
	Flexion, in degrees, median (IQR)		110	126 (113-131)
	Extension, in degrees, median (IQR)		110	0 (-2-4)

Abbreviations: IQR = interquartile range, kg = kilograms, m = meters* unless indicated otherwise, † lower scores represent unfavorable outcomes.

### KROM and observed functional status

TUG time was statistically related to age, sex and BMI, but not for knee extension or flexion (Tables 2 & 3). GAM did not suggest that adding curvilinear-terms improved the statistical model on the relationship between TUG scores and KROM (Tables 2 & 3). Therefore, no piecewise regression modeling was undertaken.

**Table 2:** Association between knee extension and self-reported and performance-based measures of functioning.

		KOOS ADL			KOOS Sport/Rec			TUG			6MWT		
Predictors		*b*	*SE*	*p*	*b*	*SE*	*p*	*b*	*SE*	*p*	*b*	*SE*	*p*
Intercept		81.12	17.23	<0.01	43.99	18.11	0.02	-5.38	3.79	0.16	930.38	126.08	<0.01
Age (years)		0.19	0.19	0.31	0.18	0.20	0.37	0.11	0.04	0.01	-3.69	1.37	0.01
Sex		-3.55	3.02	0.24	-7.20	3.18	0.03	1.44	0.67	0.03	-40.27	22.31	0.07
BMI (kg/m/m)		-1.09	0.30	<0.01	-1.12	0.32	<0.01	0.21	0.07	<0.01	-7.87	2.23	<0.01
Knee flexion (°)		0.19	0.35	0.60	0.31	0.37	0.40	0.11	0.07	0.14	-3.71	2.39	0.13
	R^2^	0.16			0.18			0.19			0.20		
	Test curvilinearity	p = 0.69*			p = 0.46*			p = 0.25*			p = 0.28*		

Abbreviations: 6MWT = Six-Minute Walk Test, ADL = Activities of Daily Living, BMI = Body Mass Index, KOOS = Knee Osteoarthritis Outcome Score, KROM = Knee range of motion, Sport/Rec = Sport and recreational activities, TUG = Timed Up and Go test.

* Chi-square test for difference between the linear fit and the regression smoother.

**Table 3:** Association between knee flexion and self-reported and performance-based measures of functioning.

		KOOS ADL			KOOS Sport/Rec			TUG			6MWT		
Predictors		*b*	*SE*	*p*	*b*	*SE*	*p*	*b*	*SE*	*p*	*b*	*SE*	*p*
Intercept		71.56	28.29	0.01	40.99	29.82	0.17	0.55	6.29	0.93	701.07	208.47	<0.01
Age (years)		0.21	0.19	0.26	0.20	0.20	0.32	0.12	0.04	<0.01	-3.87	1.35	0.01
Sex		-3.28	3.08	0.29	-7.09	3.25	0.03	1.36	0.68	0.05	-36.65	22.55	0.11
BMI (kg/m/m)		-0.99	0.35	<0.01	-1.07	0.37	<0.01	0.17	0.08	0.03	-6.25	2.61	0.02
Knee flexion (°)		0.05	0.13	0.72	<0.01	0.14	0.98	-0.04	0.03	0.15	1.54	0.94	0.10
	R^2^	0.16			0.18			0.19			0.20		
	Test curvilinearity	p = 0.57*			p = 0.88*			p = 0.17*			p = 0.06*		

Abbreviations: 6MWT = Six-Minute Walk Test, ADL = Activities of Daily Living, BMI = Body Mass Index, KOOS = Knee Osteoarthritis Outcome Score, KROM = Knee range of motion, Sport/Rec = Sport and recreational activities, TUG = Timed Up and Go test.

* Chi-square test for difference between the linear fit and the regression smoother.

**Figure 1:**
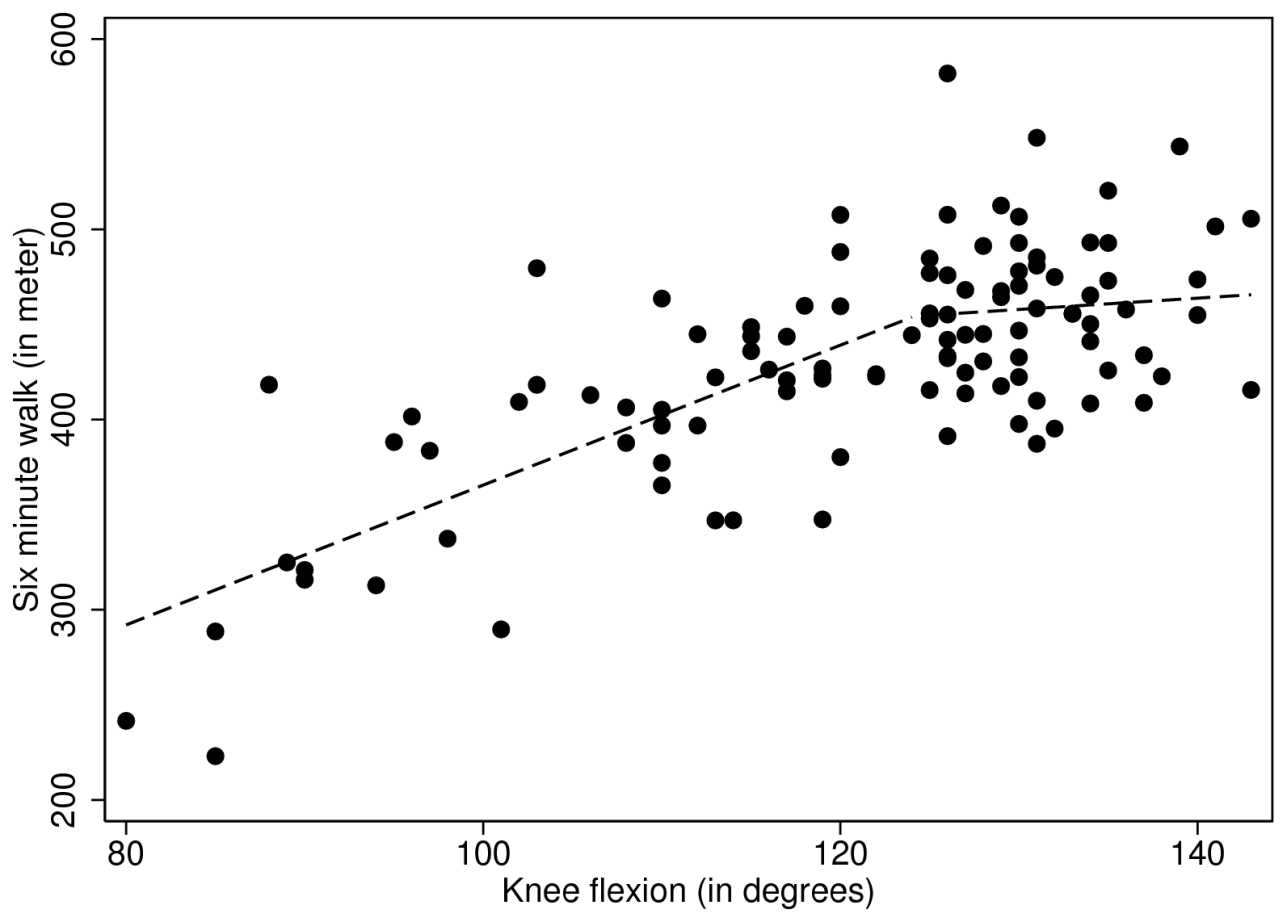
Piece-wise regression for six minute walk (in meters) and knee flexion (in degrees).

Linear regression modeling demonstrated a relationship between 6MWT distance and age, sex and BMI, but, again, not for knee flexion or extension. Statistical testing of the linear and the regression smoother lines did not indicate that adding curvilinear-terms impacted the statistical relationship between 6MWT and knee extension. However for 6MWT distance and knee flexion, GAM did hint towards a curvilinear relationship (p = 0.06). A two-segment piecewise regression model, with intersections at 125° flexion estimated slopes of 2.68 (95%-Confidence Interval [CI] 0.42-4.93) and -5.96 (95%-CI -12.81-0.90). Adding this intersection to the regression model improved the fit model (R^2^ = 0.221) compared with the base model (R^2^ = 0.199) (Figure 1).

### KROM and self-reported functional status (composite scores)

*KOOS subscale daily activities* was significantly associated with BMI, but not with age, sex or knee extension or flexion (Tables 2 & 3). GAM did not indicate that the addition of curvilinear-terms significantly impacted the relationship between self-reported daily activities and KROM. Therefore no piecewise linear modeling was undertaken.

Significant associations were found between *KOOS subscale sports and recreational activities* and sex and BMI, but not for age, knee extension or knee flexion (Tables 2 & 3). Adding locally weighted regression smoothers did not statistically alter the association between the adjusted self-reported sports and recreational activities and flexion or extension (Tables 2 & 3); therefore not warranting further piecewise linear modeling.

### KROM and self-reported independent activities (item scores)

Logistic modeling yielded no statistically significant associations between KROM extension and the 19 daily, sports and recreational activities, therefore not warranting further inspections of their specificity and sensitivity. For knee flexion, logistic modeling yielded two daily activities that were associated with reduced knee flexion; 1) having severe or extreme difficulty standing (p = 0.005) and 2) having severe or extreme difficulty picking up objects from the floor (p = 0.068); however the magnitude of the AUC (0.681 and 0.597, respectively) did not warrant further investigation regarding their specificity and sensitivity.

## Discussion

The objective of this study was to investigate both the association between KROM and different measures of physical functioning utilizing linear- and curvilinear-terms. Data from our convenience sample of people with knee OA suggest that there is not a statistical (either linear or curvilinear) or a clinical relationship between KROM (neither flexion nor extension) and self-reported or observed measures of physical functioning.

To our knowledge, this is the first study that describes the association between KROM and functional ability in-depth, because previous studies on this topic only make mention of linear regression techniques. Our finding that a statistical association between KROM and functioning is lacking is consistent with the data of Holla et al (2010) who did not find a relationship between KROM and activity limitations (as measured with help of the WOMAC questionnaire) in their cohort study [2]. Yet other researchers did report statistical associations between KROM and self-reported (β ranging from 0.12 to 0.20) and/or observed functioning (β ranging from 0.04 to 0.30) [4–6,18]. One might argue that these differences occurred through differences in KROM between studies. However, KROM scores of the people included in this study where either comparable or worse than previous studies [2,4–6]; an interesting finding in itself. Nonetheless, considering that most of these associations are fairly weak, we believe that the time invested on merely improving knee flexibility in people with knee OA to improve physical function might - if even considered - be targeted to a relative small subsample of the population that represent the far left side of Figure 1. And in case of all others, this time should better be invested on alternative treatment priorities; a conclusion also supported by post-arthroplasty data [19–21].

Our data namely suggests that there might be a threshold at 125 degrees of knee flexion, after which more knee flexion does not add to more gains in walking distance as measured with the six-minute walk test. Biomechanical experiments have demonstrated that joint restrictions result in slower walking, less covered distance and greater energy expenditure [22,23], however evidence shows that the knee normally only flexes up to 70 degrees during fast gait [24]. Therefore, we consider the finding that ROM up to 125 degrees contributes significantly to six-minute walk test distance potentially spurious. In addition, even if this cut-off is a true finding, the magnitude of the effect is only small.

Strengths of this study include the low number of missing values, inclusion of both self-report and observed measures of physical functioning, and the in-depth statistical analysis. However some limitations also need to be addressed. By including a convenience sample of people with OA who were awaiting TKA, we might have introduced some sampling bias. After all, people enrolled in clinical trials rarely represent the general population [25]. Furthermore, we only studied the knee of the involved side and neglected the uninvolved side. Because one of the selection criteria for the studies was minimal contralateral knee OA, it was believed that this would add little to the studied associations, while perhaps over-fitting the statistical model. Future studies should consider evaluating chain mechanisms by incorporating measures of hip and ankle flexibility in these curvilinear analyses as well.

## Conclusions

In conclusion, our data do not suggest that in this study sample KROM is associated with measures of objective and self-reported physical functioning in people with knee OA scheduled for TKA surgery. The lack of a (curvi)linear association between knee mobility and physical functioning suggests a limited role for ROM exercises when improving functioning in individuals with knee OA. However, it does not mean ROM should be disregarded completely in the management of OA [26], as it still plays, for instance, an important role in the diagnosis of OA [27].
